# Delirium in psychiatric settings: risk factors and assessment tools in patients with psychiatric illness: a scoping review

**DOI:** 10.1186/s12912-024-02121-6

**Published:** 2024-07-08

**Authors:** Cheng Huang, Bei Wu, Haiqin Chen, Hong Tao, Zhuqin Wei, Liming Su, Lina Wang

**Affiliations:** 1 School of Medicine, Huzhou University, 759 Second Ring Road East, Huzhou, Zhejiang 313000 China; 2 Health Management Center, Deyang People’s Hospital, Deyang, Sichua 618000 China; 3 Rory Meyers College of Nursing, New York University, New York, NY USA; 4 Nursing Department, Huzhou Third People’s Hospital, Huzhou, Zhejiang 313000 China; 5 AdventHealth Whole-Person Research, Orlando, FL USA

**Keywords:** Delirium, Dementia, Identification, Psychiatry, Risk factors, Scoping review

## Abstract

**Background:**

Delirium is a common disorder affecting patients’ psychiatric illness, characterized by a high rate of underdiagnosis, misdiagnosis, and high risks. However, previous studies frequently excluded patients with psychiatric illness, leading to limited knowledge about risk factors and optimal assessment tools for delirium in psychiatric settings.

**Objectives:**

The scoping review was carried out to (1) identify the risk factors associated with delirium in patients with psychiatric illness; (2) synthesize the performance of assessment tools for detecting delirium in patients with psychiatric illness in psychiatric settings.

**Design:**

Scoping review.

**Data sources:**

PubMed, Web of Science, and Embase were searched to identify primary studies on delirium in psychiatric settings from inception to Dec 2023 inclusive. Two independent reviewers screened eligible studies against inclusion criteria. A narrative synthesis of the included studies was conducted.

**Results:**

A final set of 36 articles meeting the inclusion criteria, two main themes were extracted: risk factors associated with delirium in patients with psychiatric illness and assessment tools for detecting delirium in psychiatric settings. The risk factors associated with delirium primarily included advanced age, physical comorbid, types of psychiatric illness, antipsychotics, anticholinergic drug, Electroconvulsive therapy, and the combination of lithium and Electroconvulsive therapy. Delirium Rating Scale-Revised-98, Memorial Delirium Assessment Scale, and Delirium Diagnostic Tool-Provisional might be valuable for delirium assessment in patients with psychiatric illness in psychiatric settings.

**Conclusions:**

Delirium diagnosis in psychiatric settings is complex due to the overlapping clinical manifestations between psychiatric illness and delirium, as well as their potential co-occurrence. It is imperative to understand the risk factors and assessment methods related to delirium in this population to address diagnostic delays, establish effective prevention and screening strategies. Future research should focus on designing, implementing, and evaluating interventions that target modifiable risk factors, to prevent and manage delirium in patients with psychiatric illness.

**Supplementary Information:**

The online version contains supplementary material available at 10.1186/s12912-024-02121-6.

## Abstract (significance)

**What is already known about the topic?:**

• Delirium is common among individuals living in psychiatric settings and remains clinically under-detected, and it is associated with the length of hospital stay and poor prognoses.

• Dementia with behavioral and psychological symptoms of dementia (BPSD) and other psychiatric illnesses has been shown to make delirium more likely. However, despite extensive research on factors associated with delirium, there is a notable lack of studies conducted in psychiatric settings.

• Early detection and identification of delirium are crucial in psychiatric settings; however, literature regarding the assessment and identification of delirium has not been synthesized, particularly in the context of comorbid psychiatric illness.

**What this paper adds?:**

• Risk factors associated with delirium in patients with psychiatric illness primarily include advanced age, physical comorbid, types of psychiatric illness, antipsychotics, anticholinergic drug, Electroconvulsive therapy (ECT), and the combination of lithium and ECT.

• Delirium Rating Scale-Revised-98, Memorial Delirium Assessment Scale, and Delirium Diagnostic Tool-Provisional might hold promising value as assessment tools for delirium in patients with psychiatric illness in psychiatric settings.

**Supplementary Information:**

The online version contains supplementary material available at 10.1186/s12912-024-02121-6.

## Introduction

Delirium as defined by the DSM-5-TR [1], is a disturbance in consciousness characterized by changes in several mental functions, grouped into two primary items: disruptions in attention (reduced ability to direct, focus, sustain, and shift attention) and awareness (accompanied by reduced awareness of the environment), and disturbances in additional cognitive functions. These disturbances typically develop over a short period and tend to fluctuate in severity throughout the day. The initial prevalence of delirium among hospitalized psychiatric patients was underestimated at about 15%, limited by the capabilities of early identification methods. Subsequent research across a variety of psychiatric settings (psychiatric units/clinics within general hospital and psychiatric hospitals, geriatric psychiatry hospitals or community mental health centers) revealed higher delirium prevalence rates: 19% in psychiatric outpatient memory loss clinic, 18.2% in the mental health clinics, 33.6% in psychiatric critical care unit, and 14.6% in psychiatric inpatients settings [2–5]. Additionally, in some countries like Ireland, Japan, and China, etc., patients with dementia may receive care in psychiatric units within general hospitals, specialized geriatric psychiatric units, or psychiatric hospitals, with admission rates from 26.9%~41.73% [6–8]. These settings prioritize specialized care for older adults with dementia, addressing BPSD or/and other psychiatric illnesses. The prevalence of delirium in patients with dementia was found to be 5% in the geriatric psychiatry clinic and 19.4% in the memory clinic of a psychiatric hospital [9, 10]. Delirium is associated with significant adverse outcomes, including functional dependence, institutionalization, cognitive impairment, dementia, and mortality [11]. Moreover, the standardized mortality ratio (SMR) of delirious psychiatric inpatients has higher mortality than psychiatric inpatients in general (SMR = 1.7) [12]. Nonetheless, delirium often goes unnoticed or misdiagnosed in psychiatric settings, with nearly 50% of cases being missed [13]. This is primarily due to its fluctuating nature and overlapping symptoms with other psychiatric illnesses, leading to “diagnostic overshadowing”, where clinical staff attribute the signs and symptoms of delirium to a pre-existing psychiatric illness [13].

**Fig. 1:**
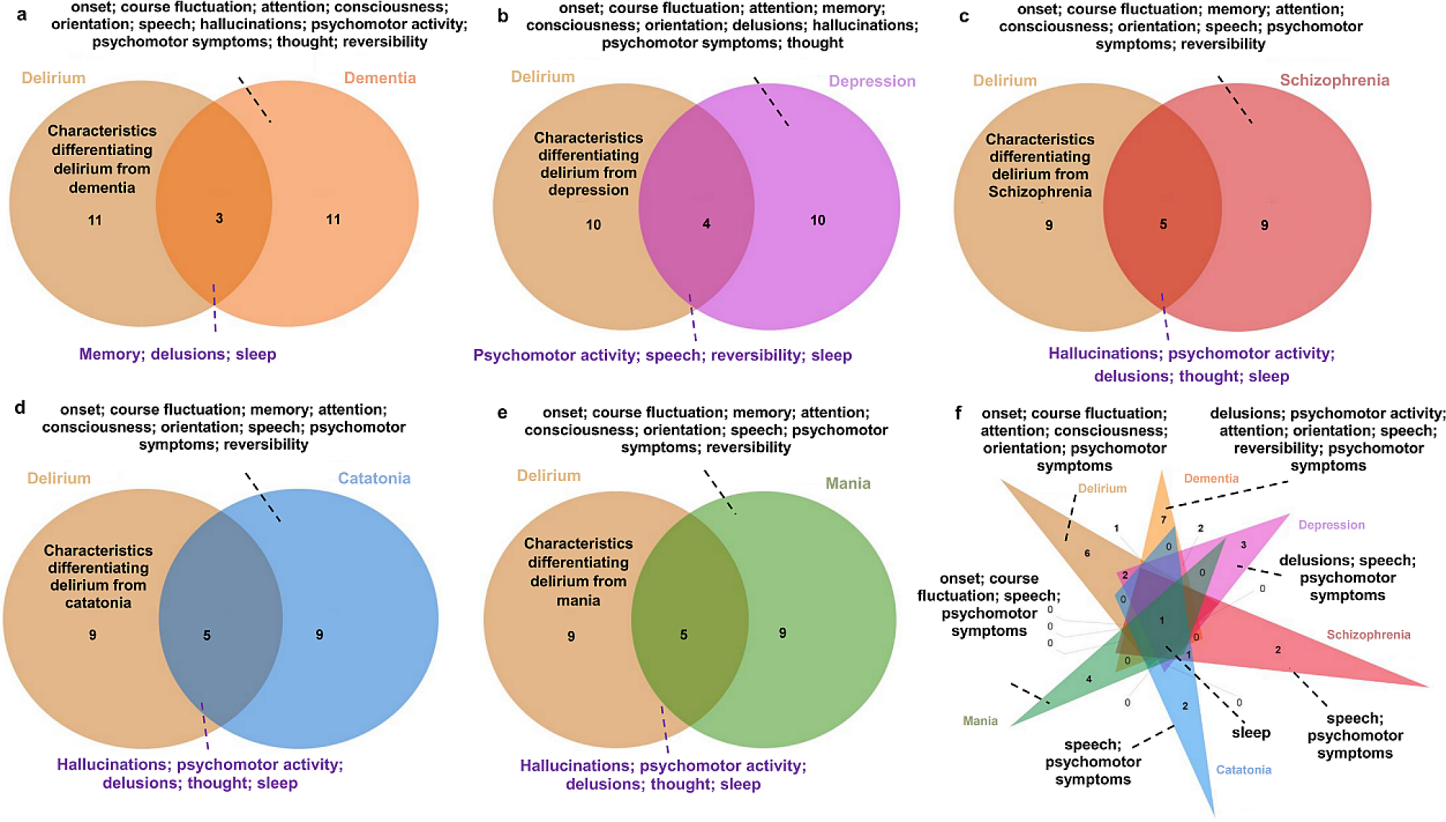
Veen diagram of overlap symptoms between delirium and psychiatric illnesses

Delirium shares many overlapping symptoms and features with psychiatric illnesses, including dementia, depression, schizophrenia, catatonia, mania, such as hallucinations, bizarre delusions and disorganized thinking, complicating the differentiation of these conditions (seen as Fig. 1). Delirium is distinguished by the rapid onset of symptoms, which emerge and fluctuate rapidly within hours or days. Acute disturbance in attention is a hallmark feature of delirium, which can manifest in various attentional domains, and another key feature is disturbance in thought clarity [14]. Notably, the term ‘disorders of consciousness’ is often used as an umbrella term for diagnosing delirium. Consequently, expression like disturbance in awareness, impaired consciousness, and thought disorganization are commonly used across different diagnostic systems to describe impaired thought clarity [14], leading to considerable confusion. Actually, awareness involves clear and coherent information processing, assessed through narrative responses or by observing a patient’s engagement with the environment. Disturbance in awareness captures the qualitative aspect of consciousness, ranging from clear to clouded [14]. In contrast, the quantitative aspect of consciousness (i.e. “reduced” as in “reduced arousal”), often fails to reflect the clarity of thought content and is typically used to evaluate the level of cerebral arousal [14, 15]. This helps distinguish delirium from coma, where there is no cerebral arousal or consciousness, and thus no mental functions. In delirium, however, the cerebral cortex is aroused with altered higher-level mental functions [15]. Additionally, altered level of activity describes a third core feature of delirium, including motor and sleep/wake cycle disturbances [14]. Fluctuating state of delirium also overlap with other psychiatric illnesses, including memory disturbances, disorientation, perception disturbances, and emotional lability [16, 17].

Delirium, distinct from dementia (except dementia with Lewy bodies characterized by fluctuating arousal) and depression, occurs suddenly with disturbance of consciousness, short attention span lacking in direction and selectivity and easily distracted, fluctuates throughout the day, and is usually worse at night [17, 18]. In contrast, dementia is characterized by relative preservation of the level of consciousness, attention, and orientation until quite late in the illness progression [19]. In the early stage of dementia, attention is usually unaffected (except in late stage), signs and symptoms manifest as progressive memory loss and an inability to perform new tasks or retain new information [17]; subsequently, expressive aphasia (in severe dementia), the inability to carry on complex conversations, emotional instability, reduced attention to complex things, and disturbed sleep-wake cycles (day/night reversal, wandering at night and fragmented in severe dementia) may arise as the disease progresses [18]. Depression is mainly characterized by low mood and/or loss of interest in most activities [16], often preserving language skills and the ability to learn new information; symptoms include sleep pattern changes (especially at the end of the sleep cycle), less energy, difficulty concentrating, and memory loss [18]. In schizophrenia, patients commonly experience verbal auditory hallucinations, less distinct visual hallucinations, level of arousal and no inattention, often with a psychiatric history [20]. Catatonia is a syndrome marked by its prominent motor, behavioral, cognitive and affective abnormalities, characterized by stupor (lack of movement and response), mutism (inability to speak), posturing, and agitation [21]. Notably, there is significant overlap with delirium, as both conditions exhibit hypoactive and hyperactive features. Hypoactive delirium is characterized by reduced motor activity, speech, and behavioral withdrawal, closely resembling catatonia. Similarly, impulsivity, mannerisms, excitement, and combativeness observed in catatonia are also reminiscent of hyperactive delirium [22]. Mania characterized by elevated mood, racing thoughts, and increased goal directed activities. It’s worth noting that patients with mania may also experience co-occurring delirium, described as delirious mania, marked by inattention, disorientation, altered consciousness, psychomotor abnormalities, and distorted thought process [20]. Additionally, EEG patterns (delirium typically manifests with generalized slowing in brain activity, featuring prominent delta and theta waves, reduced alpha waves, and fluctuating patterns [23]) and the underlying cause can aid in distinguishing delirium from psychiatric illnesses.

Additionally, delirium subtypes can be further identified based on disturbance in cognitive (attention, orientation, visuospatial capacity, memory), qualitative thought clarity (course of thought, language and comprehension), and quantitative activity level, including motor activity and sleep/wake cycles, reflecting delirium’s myriad clinical expressions [14]. These domains allow for the classification of delirium into hyperactive, hypoactive, mixed subtype [1]. Furthermore, delirium free of a motor subtype has also been identified [24]. These delirium subtypes exhibit much heterogeneity in the manifestation of clinical features in patients with psychiatric illness. Hyperactive delirium, characterized by increased motor activity, restlessness, agitation, aggression, wandering, hyper alertness, hallucinations and delusions, and inappropriate behavior, which is more detectable with a better prognosis, can easily cause individuals significant distress due to confusion about their surroundings and time [25]. However, the similarities between hyperactive delirium and the symptoms of schizophrenia and mania make it easily confused and ignored [26]. Conversely, hypoactive delirium is characterized by reduced motor activity, lethargy, withdrawal, drowsiness and staring into space, frequently goes undetected or is misdiagnosed as depression, leading to the highest mortality rate and a poorer prognosis [27]. Mixed delirium has a tremendous symptom burden with high levels of perceptual disorders, delusions, and agitation, with a relatively poor prognosis [25]. Delirium free of a motor subtype has less burden of symptomatology, less severity, and a better prognosis [27]. The above heterogeneity of delirium subtypes increases the difficulties of delirium identification. At times, psychiatric syndromes can even co-occur with delirium e.g., catatonia or delirious mania (characterized by a rapid onset of delirium, mania and psychosis), introducing further clinical complexity [20]. Delayed recognition and management have deleterious effects on morbidity, length of stay in hospital, and mortality. Furthermore, depending on the severity and etiology, patients may experience difficulty controlling emotions and psychotic symptoms that are not essential for the delirium diagnosis. This may include subsyndromal delirium, a milder form within the syndrome continuum of delirium, sharing the same symptoms affecting higher cerebral cortical functions, such as disturbances in attention, higher-level thought, and circadian rhythm in which the severity of cognitive impairment falls short of that required for a delirium diagnosis [1, 15, 28, 29].

Similar to other psychiatric conditions, delirium involves abnormalities in various higher cerebral cortical functions, and its wide range of symptoms complicates differentiation from other psychiatric disorders. Therefore, this speaks to the need for better assessing the symptoms of delirium, searching for its causes, and selecting appropriate clinical assessment methods or tools to facilitate an accurate diagnosis of psychiatric patients (including dementia) with suspected delirium.

The emphasis of delirium identification should be put on effective delirium-related risk factors screening. The etiology of delirium is commonly multi-factorial, characterized by a complex interaction between predisposing factors like baseline vulnerability due to older age, dementia, cognitive impairment, depression, and functional disabilities and precipitating factors often stemming from hospital interventions such as medication use, physical restraints, iatrogenic events, and infections; generally, the more predisposing factors that are present, the fewer precipitating factors that are needed [30]. Although risk factors for delirium have been identified in other health care institutions, little research evaluation the risk factors of delirium in psychiatric settings. Furthermore, delirium-related risk factors may contribute differently to the psychiatric settings and other health care institutions [4]. A systematic review and meta-analysis revealed that age, pain while resting, malnutrition, acute infections, antipsychotics, and antibiotics confer a risk of delirium in older adults with dementia but not specifically with other psychiatric patients [31]. A past psychiatric history, dementia, depression, or other psychiatric illness, has been shown to make delirium more likely [13, 32]. However, potential risk factors associated with the development of delirium in patients with psychiatric illness in psychiatric settings are less known.

Delirium screening in psychiatric settings can and should be advocated, but the currently available tools may not be adequate to achieve the desirable level of accuracy, which may be another contributing factor for delirium being unnoticed or misdiagnosed in psychiatric settings. Selecting a delirium detection tool with high sensitivity and specificity in psychiatric settings is essential and, where possible, has been validated in the same clinical setting. Although the effectiveness of delirium assessment tools has been most validated in the emergency department, intensive care unit, and general inpatient unit [32–34], there has been minimal evaluation of the effectiveness of delirium assessment tools in patients with psychiatric illness in psychiatric settings.

Addressing the above knowledge gaps will help to provide a more robust evidence base to inform ongoing efforts for effective prevention, detection, and management of delirium in psychiatric settings. Therefore, a scoping review was selected as appropriate to map the existing literature while not restricting included articles to some methods or quality criteria. This scoping review aimed to review the discoveries in delirium presented in the literature to identify the risk factors associated with delirium in patients with psychiatric illness, and summarize the current assessment tools to facilitate the detection of delirium in psychiatric settings.

## Methods

The scoping review methodological framework proposed by Arksey and O’Malley comprises five distinct stages [35]. The five steps are as follows: (i) identifying the research questions; (ii) identifying relevant studies; (iii) selecting the studies; (iv) charting the data; and (v) collating, summarizing, and reporting the results. This review was conducted following Preferred Reporting Items for Systematic reviews and Meta-Analyses extension for Scoping Reviews (PRISMA-ScR) [36].

### Stage 1: Identifying the research question

Identifying the research question is the first step leading the search strategies for this literature review. The research questions for this literature search were: (1) “What are the risk factors associated with delirium in patients with psychiatric illness in psychiatric settings?” (2) “What are the assessment tools for delirium in psychiatric settings?”

### Stage 2: Identifying the relevant studies

In consultation with a university health sciences librarian, a search strategy was developed to identify relevant studies. Search terms included medical subject headings (MeSH) and keywords, i.e., “delirium”, “mental disorders”, “dementia”, “psychiatric department, hospital”, “psychiatric patient/psychiatric patients/ psychiatric department/ psychiatric ward”. The search was conducted across three electronic databases: PubMed, Web of Science, and Embase, covering the period from inception to Dec 2023, in accordance with the established search strategy (see Table 1 for the complete search strategies for PubMed). In addition, a manual search of the references of extracted articles was conducted to identify studies not captured in the electronic database searches. The language was limited to English.

**Table 1:** Search strategies

Search strategy: example for PubMed	
Number	Search items
1	**MeSH Terms**: “Mental Disorders“[Mesh] OR Mental Disorder OR Psychiatric Illness OR Psychiatric Illnesses OR Psychiatric Diseases OR Psychiatric Disease OR Mental Illness OR Illness, Mental OR Mental Illnesses OR Psychiatric Disorders OR Psychiatric Disorder OR Behavior Disorders OR Diagnosis, Psychiatric OR Psychiatric Diagnosis OR Mental Disorders, Severe OR Mental Disorder, Severe OR Severe Mental Disorder OR Severe Mental Disorders
2	**MeSH Terms**: “Psychiatric Department, Hospital“[Mesh] OR Psychiatric Departments, Hospital OR Department, Hospital Psychiatric OR Departments, Hospital Psychiatric OR Hospital Psychiatric Departments OR Hospital Psychiatric Department
3	**MeSH Terms**: “Dementia“[Mesh] OR Dementias OR Amentia OR Amentias OR Senile Paranoid Dementia OR Dementias, Senile Paranoid OR Paranoid Dementia, Senile OR Paranoid Dementias, Senile OR Senile Paranoid Dementias OR Familial Dementia OR Dementia, Familial OR Dementias, Familial OR Familial Dementias
4	**Title/Abstract**: Psychiatric patients OR psychiatric patient OR psychiatric department OR psychiatric ward OR psychiatr*
5	**Title/Abstract**: Deliriums OR delirious OR delirium
6	**Title/Abstract**: Intensive care unit (ICU) OR critical care unit (CCU) OR Emergency Intensive Care Unit (EICU) OR Pediatric Intensive Care Unit (PICU) OR post-ICU OR Postoperative OR Surg* OR Emergency OR Acute OR palliative patient OR hip fracture OR critically ill patient OR general hospital OR withdrawal delirium OR alcohol OR Delirium tremens
7	1 OR 2 OR 3 OR 4
8	5 AND 7 NOT 6

### Stage 3: Study selection

Following the completion of all database searches, the citations were compiled and entered into EndNote bibliographic manager, where any duplicated citations were removed. Titles and abstracts were independently screened by two reviewers (HC and WZQ) using the inclusion criteria noted below. The full-text screening was then undertaken following the same process. Any disputes were discussed, and consensus was reached between the reviewers. Should dispute resolution not have been achieved, a third expert reviewer (WLN) would have been consulted.

Study selection was guided by pre-determined inclusion and exclusion criteria. Studies were eligible for inclusion if they were: (1) addressed the target question -- focused on delirium risk factors for patients with psychiatric illness, whether such patients are admitted to a psychiatric ward/unit, memory clinic, mental health clinic, or psychiatric critical care unit, but excluded delirium secondary to other non-psychiatric conditions; and assessment tools for delirium in psychiatric settings; (2) publications written in English; (3) articles were peer-reviewed. Editorials, commentaries, letters, abstracts, and not peer-reviewed were excluded. Studies involving patients with dementia extracted from long-term care facilities and nursing home were also excluded due to significant differences in treatment environments and methods/interventions compared to psychiatric settings.

### Stage 4: Charting the data

The data charting took into consideration the following information and was entered into an excel spreadsheet, including the author(s), year of publication, study location, the purpose of the study, methodology (study design, sample), and key findings of studies.

### Stage 5: Collating, summarizing, and reporting the results

After summarizing the study characteristics to provide an overview of the studies included, this study explored the risk factors of delirium, and assessment tools for delirium in patients with psychiatric illness in psychiatric settings.

## Results

### Identification and selection of relevant studies

**Fig. 2:**
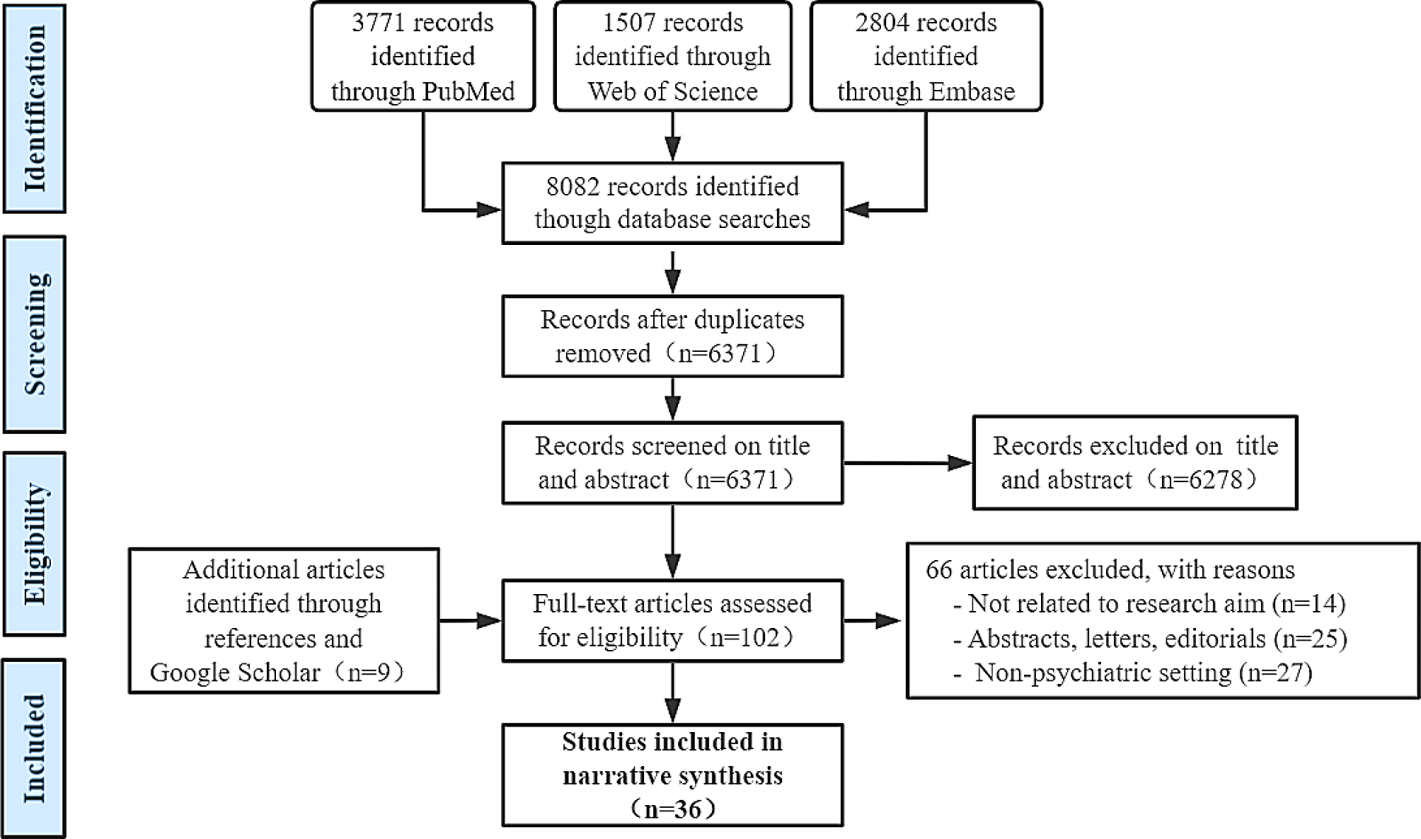
Overall flow of scoping review search and selection

The detailed process of search and selection of studies was illustrated using the PRISMA flow chart presented in Fig. 2. A total of three electronic databases produced 8,082 records, with nine additional records identified through reference lists of included articles and Google Scholar. Among these, 1,711 duplicates were removed, and 6,278 articles were excluded after screening titles and abstracts. A total of 102 full-text papers were assessed and screened for eligibility. A further 66 were removed after the inclusion criteria were matched, leaving 36 papers in this scoping review.

### Characteristics of included studies

Supplemental Material 1 provides a summary of the included studies. Of the 36 articles included in this study, 32 were research studies (i.e., utilizing cross-sectional, cohort, longitudinal study, case studies, case-control studies, diagnostic studies, and randomized controlled studies), and 4 were review articles.

Included studies originated from various countries, with 12 papers undertaken in the United States of America (USA), 4 in Canada, 3 in China, and Korea, 2 in United Kingdom (UK), Japan, and Spain, and 1 each in Italy, Ireland, Netherlands, India, Nigeria, Australia, Germany, and Portugal. 26 papers presented risk factors of delirium related to psychiatric settings, and 10 introduced the assessment tools for delirium in patients with psychiatric illness. According to the existing studies, this scoping review identified populations with psychiatric illness in psychiatric settings primarily including dementia, schizophrenia, bipolar disorder, anxiety disorders, depressive disorders, personality disorders, and manic episodes.

### Potential risk factors of delirium in patients with psychiatric illness in psychiatric settings

The factors linked to delirium in patients with psychiatric illness are in three categories: socio-demographic characteristics, medical conditions, and iatrogenic factors. Table 2 shows the determinants associated with delirium and their frequency mentioned in the included studies.

1. The risk factors identified related to delirium were advanced age [2, 3, 16, 37–40], being female [40], and marital status (married) [4], regarding the socio-demographic characteristics.
2. Medical conditions related to the onset of delirium, including prior delirium [2, 39], infections [2, 5, 17, 18], adverse effects of medication [41], urinary tract infection [18, 42], physical disability [19, 39], physical comorbid [4, 5, 38], diagnosis of bipolar affective disorder or other psychosis [4, 38, 43], and the total sleep time [44], and malnutrition [20].
3. Iatrogenic factors were categorized and summarized according to whether patients received medication, catheterization, and other factors. The type of pharmacological treatment associated with delirium include non-psychotropic medication (anticholinergic drug and antibiotics) and psychotropic medication [45]. Psychotropic medication associated with delirium were antipsychotics [5, 17, 37, 38, 46], antidepressants [17, 47, 48], mood stabilizer [37], and no use of benzodiazepines [39]. Specifically, antipsychotics strongly associated with delirium, was olanzapine [48]. Antidepressants associated with delirium include, duloxetine and bupropion [47], and tricyclic antidepressant drugs [48]. Mood stabilizer associated with delirium was lithium [37]. Non-psychotropic drugs associated with delirium were anticholinergic drug [37, 48, 49], antibiotics [46, 50], antiparkinsonian [3, 37]. The catheterization correlated with delirium were intravenous catheters and urinary catheters, which have also been associated with the risk factors for hypoactive delirium [46]; when combined with antipsychotics and antibiotics, they have been verified to be related to the incidence of mixed delirium. In addition, urinary catheters and antipsychotics were associated with hyperactive delirium [46]. Finally, this study also identified other factors, like ECT [51], and the combination of lithium and ECT [52, 53].

**Table 2:** Frequency of mentioned risk factors in included studies for delirium among patients with psychiatric illness in psychiatric settings

Socio-demographic characteristics			Medical conditions								
Age	Female gender	marital status (married)	Prior delirium	Infections	Adverse effects of medication	Urinary tract infection	Physical disability	physical comorbid	Psychiatric illness diagnosis	Sleep time	malnutrition
**+** **+** **+** **+** **+** **+** **+** ^a^	**+**	**+** ^b^	**+** **+** ^a^	**+** **+** **+** ^a^ **+** ^c^	**+**	**+** **+**	**+** **+**	**+** **+** **+** ^b^ **+** ^c^	**+** **+** **+** ^b^	**+**	**+**
				**Iatrogenic factors**							
			**Medications**				**Catheterization**		**Others**		
**psychotropic drugs**				**Non-psychotropic drugs**							
**Antipsychotics**	**Antidepressants**	**Mood stabilizer**	**No use of benzodiazepines**	**Anticholinergic drug**	**Antibiotics**	**Antiparkinsonian**	**Intravenous catheters**	**Urinary catheters**	**ECT**		
**+** **+** **+** **+** **+** ^c^	**+** **+** **+**	**+**	**+**	**+** **+** **+**	**+** **+**	**+** **+**	**+**	**+**	**+** **+** +* +*		

*Note* The “+ symbols” refers to the risk factors have been mentioned in the included studies, and the number of “+ symbols“ represents the number of times the same risk factor was mentioned in the included studies

* Combination of lithium and ECT was associated with delirium

^a^ Represents risk factors for delirium in the memory clinic of a psychiatric hospital

^b^ Represents risk factors for delirium in patients with psychiatric illness in psychiatric critical care unit

^c^ Represents risk factors for delirium in mental health clinic

### Assessment tools for delirium in psychiatric settings

**Fig. 3:**
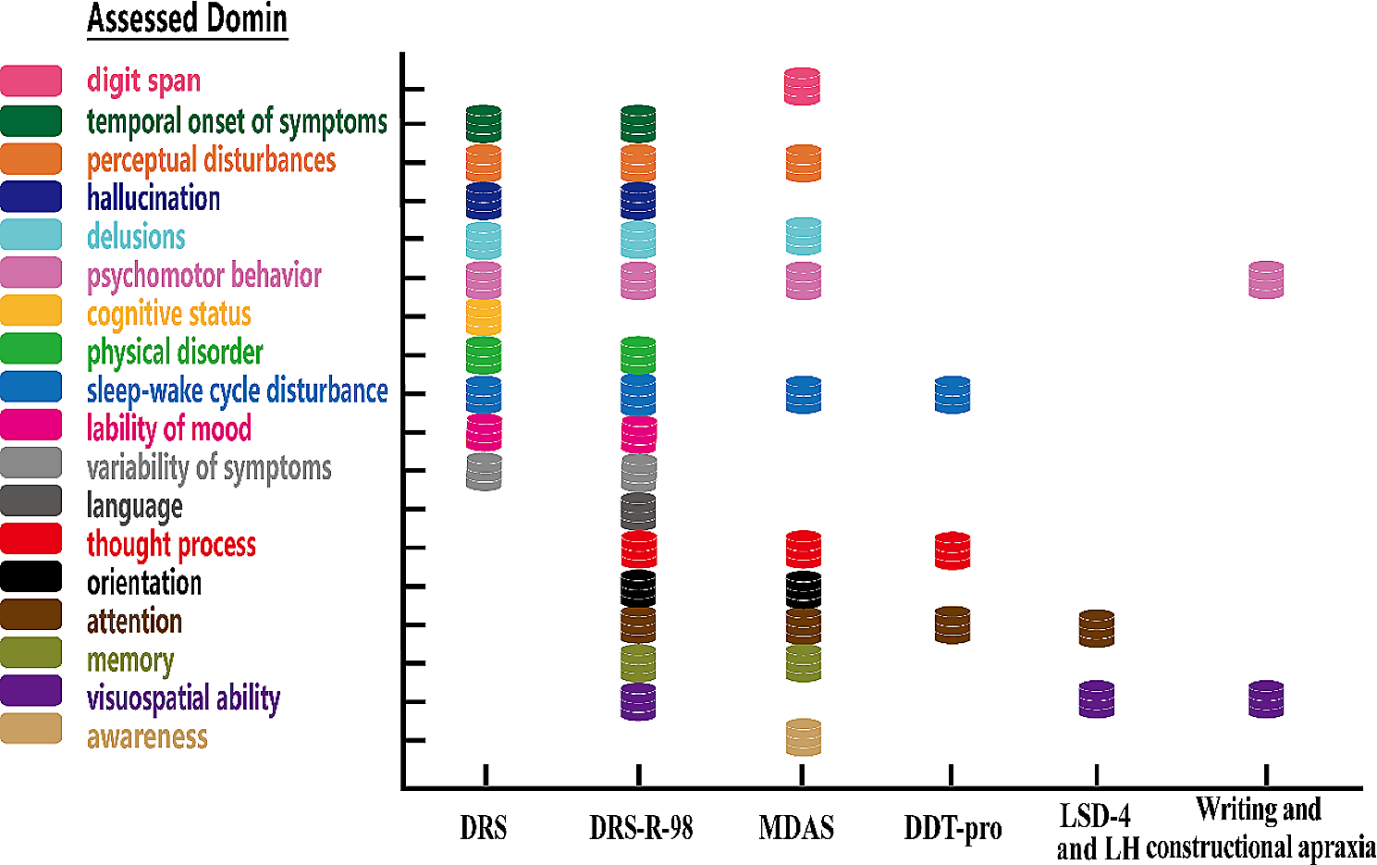
Delirium assessment tools and its assessed domains in psychiatric settings

Table 3 provides a summary of the delirium assessment tools used in psychiatric settings, as explored in the included studies, including assessed items, sensitivity/ specificity, cutoff value, and test time, etc., and Fig. 3 shows the details for assessment tools and its domains in delirium in psychiatric settings.

**Table 3:** Performance of assessment tools for delirium in psychiatric settings to discriminate delirium from other psychiatric illnesses

Test	Test time (min)	Total score	Cut-off score	Sensitivity	Specificity	Comprised items	Reference
DRS	-	32	≥ 10	94%	82%	Temporal onset of symptoms; perceptual disturbances; hallucination type; delusions; psychomotor behavior; cognitive status; physical disorder; sleep-wake cycle disturbance; lability of mood; variability of symptoms	Rosen et al., 1994; Trzepacz et al., 1988
DRS-R-98	-	46	14.5 ~ 20	89%~98%	84%~97%	Sleep-wake cycle disturbance; perceptual disturbances and hallucinations; delusions; lability of affect; language; thought process; motor agitation; motor retardation; orientation; attention; short-term memory; long-term memory; visuospatial ability; temporal onset of symptoms; fluctuation of symptom severity; physical disorder	Trzepacz et al., 2001; Huang et al., 2009; Kato et al., 2010; Lee et al., 2011; de Negreiros et al., 2008
MDAS	10	30	10	100%	100%	Awareness; orientation; memory; digit span, attention; thinking; perceptual disturbance; delusions; psychomotor activity; sleep-wake cycle	Matsuoka et al., 2001
Writing and constructional apraxia test	-	Dysgraphia: -constructional apraxia: 10	Dysgraphia: -constructional apraxia: ≤8	67.7%	84.6%	Visual perception; motor skills	Baranowski et al., 2000
DDT-Pro	-	9	≤ 7	100%	82.4%	Comprehension; vigilance; sleep-wake cycle	Kim et al., 2022
LSD-4 and LH	LSD-4: 1–2; LH: -	LSD-4: 4; LH: -	LSD-4: ≤3; LH: -	95%	43%	attention/vigilance; visuospatial abilities	Meagher et al., 2020
Machine Learning	-	-	-	77%	67%	input variables	Hercus and Hudaib, 2020

*Note* DRS/DRS-R-98 = Delirium Rating Scale, Revised Version; MDAS = Memorial Delirium Assessment Scale; DDT-Pro: Delirium Diagnostic Tool-Provisional; LSD-4 = Letter and Shape Drawing test; LH = Lighthouse (test)

#### Assessment tools for distinguishing delirium from psychiatric illnesses

1. Delirium Rating Scale/Delirium Rating Scale-Revised-98 (DRS/DRS-R-98).

In this scoping review, 5 studies validated the effectiveness and reliability of DRS and DRS-R-98 for delirium identification in psychiatric settings. As a bedside assessment tool designed by Trzepacz et al. [54], the DRS has been shown to detect delirium when assessing psychiatric patients who had a variety of psychiatric illnesses [54, 55]. However, a separate item on attention, a core symptom of delirium, was lacking. Thus, Trzepacz et al. [56] developed the DRS-R-98, which includes two sections (three diagnostic items for initial ratings and a 13-item severity scale). DRS-R98 is widely utilized in psychiatric settings due to its robust diagnostic accuracy, comprehensive assessment capabilities, and its standardized approach for differentiate delirium from dementia, schizophrenia, depression, and other psychiatric conditions [57], and it has been adapted and translated for use in several countries [58, 59].

(2) Writing and constructional apraxia test.

Writing functions evaluated by duplicated strokes or repetitive motion, angled, jagged characters or fragments, spelling errors, and missing or added words, etc. for detecting delirium [60], and the constructional apraxia was assessed by copying two intersecting pentagons (with four intersecting sides) [61]. The spelling error had a sensitivity/ specificity of 0.26/0.96 in detecting delirium in psychiatric inpatients [60]. Among older adult psychiatric inpatients, constructional apraxia had a sensitivity/ specificity of 0.68 and 0.85 in detecting delirium [60].

#### Assessment tools for distinguishing delirium from dementia

1. Memorial Delirium Assessment Scale (MDAS).

The MDAS is a ten-item score out of 30 points and takes approximately 10 min to complete. The items assess several domains of delirium, including disturbances in cognitive function (awareness, attention, orientation, memory, digit span, and disturbances in thinking), behavioral and psychomotor disturbances (perceptual disturbances, delusions and psychomotor activity), as well as sleep-wake cycle disturbances. Although the MDAS primarily focuses on assessing the severity and characteristics of delirium symptoms, Matsuoka et al., suggested that it as a valuable tool for establishing a diagnosis of delirium, effectively differentiated it from dementia or noncognitive psychiatric illnesses with a cutoff score of 10 [62]. Additionally, psychiatric evaluations, medical history reviews, and diagnostic criteria, may complement the use of MDAS for differentiation between delirium and other psychiatric disorders.

(2) Delirium Diagnostic Tool-Provisional (DDT-Pro).

The DDT-Pro is a brief structured scale comprising items on attention, circadian disturbance, and higher-level thinking. The scores range from 9 points (best performance) to 0 (worst performance). Kim et al. [63] translated the DDT-Pro into Korean and validated it in psychiatric setting. The DDT-Pro cutoff scores ≤ 6 and ≤ 7 have been shown to balance sensitivity and specificity in detecting delirium. Specifically, a cutoff of ≤ 7 is recommended for diagnosing both subsyndromal delirium and delirium, while a cutoff of ≤ 6 is more appropriate for diagnosing delirium [64], ensuring both subtle and severe forms of delirium are accurately identified, enhancing diagnostic precision.

(3) Other novel tests to identify delirium.

Another emerging approaches for detecting delirium is the Lighthouse (LH) test, which focuses on attention/vigilance, and the Letter and Shape Drawing test (LSD-4), which focuses on assessing visuospatial abilities [65]. The LH test is administered by an Android smartphone and consists of three parts: assessing whether the subject recognizes the lighthouse; assessing the focus attention through identifying the number of recognition flashes; testing the sustained attention by counting sequences of flashes. The LSD-4 consisted of a series of 4 designs that requires subjects to replicate an adjacent blank grid. Correct performance requires the subject to complete the desired shape. The reported sensitivity/overall accuracy of LSD-4 plus the sustained attention component of the LH test for distinguishing delirium was 0.95/ 0.78 [65].

Machine Learning Technology has been introduced for delirium detection. Hercus and Hudaib [13] built an accurate predictive classifier algorithm and revealed that the input variables, including age, gender (female), referral unit, psychiatric history, pain, hypoactive delirium, death, hospital stay, and the 4 “A”s Test score, were related to the delirium misdiagnosis on general hospitals. The most significant contributor to delirium misdiagnosis was a history of psychiatric illness.

## Discussion

This scoping review analyzed the content of 36 papers, and no limitation was placed on the studies’ publication year on the limited amount of research in this area. The information from this scoping review addressed the knowledge gaps in the risk factors associated with delirium, and assessment tools for delirium in patients with psychiatric illnesses in psychiatric settings. The findings of this study will provide valuable intervention clues and insights to formulate effective delirium management strategies among patients in psychiatric settings.

### Risk factors related to delirium in patients with psychiatric illnesses in psychiatric settings

The results of this study identified the complex relationship of delirium risk with demographic factors, medical conditions (e.g., pathologies), iatrogenic factors (e.g., medications, catheterization, and others). Among these risk factors, advanced age, infection, co-morbidities, and catheter use were consistent with risk factors for delirium identified in other healthcare settings. Other risk factors reported in this review, but not noted in other general settings, included psychiatric illnesses diagnosis, sleep time, psychoactive medication use, and ECT.

In the aging process, a decrease in cholinergic neurons affects brain function [66], which may be the susceptible factors for the occurrence of delirium. Infections generate inflammatory mediators, leading to the systemic release of cytokines, and activate primed microglia, leading to neuronal injury, which, in turn, leads to acute delirium [67]. Patients with psychiatric illness in psychiatric settings are more likely to receive physical restraint and catheterization due to psychological and behavioral symptoms and the lack of self-care ability in patients with severe dementia [46].

The characteristic risk factors associated with delirium in psychiatric settings in this scoping review were psychiatric illness diagnosis (especially bipolar affective disorder), psychoactive medication use (antipsychotics, antidepressants), and ECT. Bauernfreund et al., [38] demonstrated that a diagnosis of schizophrenia was associated with lower odds of in-hospital delirium diagnosis compared with bipolar affective disorder (OR = 1.66, 95%CI: 1.44–1.93). Given that the neurotransmitters hypothesis of cholinergic deficiency or dopamine excess is most frequently linked to delirium [66], the association of antipsychotic drugs, antidepressants, and mood stabilizer with delirium is not surprising. Management delirium in patients with psychiatric illness is particularly challenging, especially for those experiencing positive symptoms. In clinical practice, antipsychotics are typically the first-line pharmacological treatment used to address delirium in hospitalized patients [68]. Although the benefits of using antipsychotics may outweigh the risks for managing specific psychiatric symptoms, many studies that have found a causal relationship between antipsychotics and delirium or no therapeutic effect. Clozapine is contraindicated for delirium treatment due to its significant anticholinergic effects, which can exacerbate cognitive impairment and cause delirium [69]. Similarly, sustained-release olanzapine is potentially harmful for managing delirium because of its long-acting nature and risk of causing delirium [70]. Furthermore, study on haloperidol and ziprasidone in ICU patients has not demonstrated that antipsychotics reduce delirium duration [68]. Benzodiazepines are generally discouraged for acutely medically ill patients due to delirium risk, and there is limited evidence on their effects on key symptoms of delirium like attention deficits [71]. Additionally, current delirium assessments tend to over-emphasize hyperactive symptoms, thus, symptom relief from antipsychotic treatments in patients with hyperactive delirium might lead investigators to incorrectly conclude their general efficacy for delirium [72].

Conversely, some studies have shown that screening instruments can misclassify conditions like stupor and coma as delirium, leading to inappropriate antipsychotic treatment and incorrect identification of these medications as risk factors for delirium [73, 74]. Therefore, incorporating mechanisms like longitudinal follow-up is essential to ensure accurate diagnosis and understanding of the relationship between antipsychotics and delirium. Furthermore, antipsychotics remain widely used after ‘negative’ trials, potentially exhibiting subtype-specific responses in delirium management [75]. A large-scale multicenter prospective observational study demonstrated that antipsychotic medications are differentially effective across delirium subtypes, with hypoactive and mixed subtypes showing a more favorable response to treatment [76]. These findings emphasize the necessity for comprehensive assessment of delirium subtype and appropriate reference criteria in evaluating the safety and effectiveness of antipsychotics in delirium management.

ECT is a psychiatric treatment in which seizures are electrically induced in patients to provide relief from severe psychiatric illnesses. Delirium is a common complication following ECT, influenced by various factors including a history of cerebrovascular disease, dementia, catatonic features, impaired cholinergic function, increased seizure duration (more than 80s), and bilateral type of electrode placement [77, 78]. Additionally, a large-scale study involving 64,728 psychiatric inpatients revealed that the combination of acute course of ECT with lithium treatment increased the odds of delirium by 11.7 times compared to ECT alone, and electrode placement, lithium dose, the specific psychiatric diagnosis might influence delirium risk [52]. Therefore, reducing lithium dosage when combined with ECT may help decrease the delirium risk, a hypothesis that warrants further investigation. Noteworthy, the evolution from historic ECT to modern ECT in technology and protocols has significantly improved safety and reduced the risk of cognitive side effects, including delirium. Modern ECT utilize more precise and less intense pulse waveforms and right unilateral electrode placement, greatly reducing the risk of delirium compared to historic ECT with sine wave [79]. Moreover, customized dosages based on individual seizure thresholds, combined with enhanced safety protocols (general anesthesia and refined monitoring of vital signs and brain activity) further decrease the risk of delirium [80]. However, psychiatric staff still require close monitoring for early signs of delirium.

It is worth noting that one study investigated factors associated with subtypes of delirium and indicated that urinary catheters were significantly related to all delirium subtypes, whereas the presence of intravenous catheters was only related to hypoactive and mixed delirium subtypes. Antipsychotics correlated with mixed and hyperactive delirium, and antibiotics only with mixed delirium subtypes [46]. The relationship between antipsychotics and hyperactive delirium can be complex context-dependent. A study found that high antipsychotics usage correlates with hyperactive delirium, as antipsychotics can be prescribed to manage symptoms of agitation, aggression, or psychosis associated with hyperactive delirium [81]. However, it has also been shown that antipsychotics may lead to a transition to hypoactive delirium [82]. Some studies in different healthcare settings provide important study clues for subtype-matched risk factors for delirium in psychiatric settings. For instance, a meta-analysis on hospitalized older patients revealed that hypoactive delirium was associated with older age, poorer cognition, female gender, and increased medication use [83]. Another study conducted in ICU found that hyperactive delirium often required a delirium-targeted pharmacological strategy, mixed delirium received specific pharmacological agents (antipsychotics, α2-agonists, benzodiazepines, and propofol), and hypoactive delirium was associated with older age [84].

### Assessment tools for delirium in psychiatric settings

This scoping review identified eleven assessment tools for delirium in psychiatric settings. The selection of tools depends on multiple factors, including the time available, the level of tester skill, and the clinical settings. Discrimination of delirium from psychiatric illnesses is critical in the psychiatric settings, where despite its limitations (it requires training and psychiatric expertise), the DRS-R-98 and MDAS are most helpful. In addition, given that the co-existence of dementia and delirium is associated with worse outcomes, this scoping review also focused on available assessment tools for delirium in dementia patients. This study noted that LSD-4 plus LH have low specificity in detecting delirium in patients with dementia. These results imply a higher false positive rate, possibly due to the high prevalence of comorbid dementia and the fact that patients with dementia show impairment on the attention tests that increases with the severity of dementia, thus limiting the ability of attention tests to discriminate between dementia and delirium. This explains why attention, as a core symptom of delirium, is highly sensitive to recognizing delirium. Moreover, the results of this study noted the potential of the combination of LSD-4 and LH test in identifying delirium in patients with dementia, achieving a sensitivity of over 90% in delirium detection. Therefore, delirium and dementia can be distinguished by impaired attention and visuospatial ability. Furthermore, arousal is usually not impaired in patients with dementia, even in the advanced stages, but appears to be a specific indicator of delirium; arousal level assessment has shown promise for screening delirium superimposed on dementia (DSD). Additionally, Kim et al. [63] validated the predictive value of DDT-Pro for delirium among patients referred to the department of psychiatry, which showed that DDT-Pro appeared to be satisfactory in identifying delirium and its subtype. The sensitivity and specificity of psychiatrist evaluation of delirium were higher at a cutoff value of 6 (84% and 94.1%) and 7 (100% and 82.4%) [63]. Although these tools are available and DRS-D-98 may be among the best, their limitations highlight the necessity of comprehensive clinical evaluation to accurately distinguish diagnoses.

It is worth mentioning that some DSD detection tools were excluded from this study as they were designed and validated for non-psychiatric settings. Examples include the 4-Delirium superimposed on dementia (4-DSD) developed by Morandi et al. for patients with DSD in acute and rehabilitation hospital wards [85]; and a clinical review conducted by Priyanka et al. on the detection of DSD across care settings, including general medicine units, acute hospital and rehabilitation setting, ICU, and emergency department) [86]. However, these tools provide valuable insights for conducting research and application in psychiatric settings.

In clinical settings, delirium, dementia, and depression may exist simultaneously in the same patient and often confer increased risk for each other. Patients with delirium have 12 times greater odds of developing dementia and almost 3 times developing the risk of dose-dependent depression [87, 88], while those with dementia are 4 times more likely to develop delirium [30], and the risk of delirium in patients with depression ranges from 1.3 to 9 times higher [89]. Grover et al., found that 30.2% and 12.7%, respectively, of 205 delirium subjects had catatonia syndrome [22], presenting management challenge as catatonia responds to benzodiazepines and modified ECT [90]. However, both may also produce and exacerbate delirium, and conversely, antipsychotics used to treat symptoms of delirium may produce or exacerbate catatonia. Given the vast differences in prevention and management of these conditions, accurate recognition and labeling of these syndromes is critical. Nurse takes a crucial role in recognizing delirium, and initiating preventive measures with spending more time in direct contact with patients than any other healthcare profession. However, previous research has indicated that 85% of nurses lack confidence in screening for and identifying delirium [91], Additionally, most psychiatric nurses possess limited knowledge of delirium and do not routinely screen for it, although approximately 99% are willing to undergo training [92]. This underscores the necessity for enhanced education and structured screening procedures to improve psychiatric nurses’ ability to accurately identify delirium. Moreover, proficient observation skills within a robust therapeutic relationship empower nurses to identify delirium-related acute changes in attention or consciousness [93]. Psychiatric nursing practice should prioritize the early identification of high-risk individuals through comprehensive assessment of risk factors and symptom monitoring. Furthermore, the implementation of interdisciplinary care models, such as the Hospital Elder Life Program (HELP) for delirium management [94], may have a positive impact on patients with psychiatric illness.

## Limitations

One of the limitations of this scoping review was that the inclusion criteria limited citations to those in English only. The delirium-related determinants identified in this review are likely not the only determinants associated with delirium in people with psychiatric illness. Secondly, regarding delirium, it was included in the Diagnostic and Statistical Manual of Mental Disorders, third edition (DSM-III) under the category of “Organic Mental Disorders” in 1980. Therefore, it is possible that papers from pre-1980 period may not truly capture the concept of delirium as it is currently understood. Thirdly, no formal risk of bias assessment was performed in this study. Although that is not a methodological requirement of the scoping reviews, it does place limits on the author’s ability to comment on the robustness and rigor of the included studies. Furthermore, we cannot disregard the possibility that some studies may not have been identified despite efforts to include the relevant literature. In addition, severe eating disorders may lead to delirium due to malnutrition, dehydration, and electrolyte imbalances. Similarly, individuals with post-traumatic stress disorder (PTSD) may experience delirium under certain circumstances, such as medical triggers or medications that can induce delirium as a side effect. However, there is a lack of specific studies on the relationship between eating disorders/PTSD and delirium in the existing literature. Meanwhile, given the high prevalence of delirium superimposed on dementia and its related outcomes, studies conducted in the context of dementia were also considered, but these studies also highlighted the current lack of evidence focused on delirium in psychiatric settings. In addition, the search strategy did not include the term “encephalopathy”, potentially resulting in the omission of relevant studies pertaining to antipsychotics-induced delirium. The risk factors (including “number of times the risk factors were mentioned”) for delirium in patients with psychiatric illnesses discussed in this study are derived from included studies; caution is warranted in interpreting findings. To the best of our knowledge, this is the first scoping review exploring both risk factors and assessment tools for delirium in psychiatric settings, providing valuable insights for future research. Finally, while some case studies and epidemiological studies included in this study lack sufficient evidence to establish the mechanistic association of psychotropics with delirium, the findings still provide valuable insights and essential clues for future empirical studies.

## Future directions

This study examined the current body of knowledge in the field of delirium in psychiatric patients and revealed several promising lines of research for future exploration. More specifically, information regarding the distribution, severity, and risk factors of delirium subtypes in psychiatric patients could be used in future quantitative studies to map the characteristics of participant’s levels. Secondly, although some assessment tools could detect delirium with high sensitivity, some also presented unacceptably high false positive rates and did not evaluate the delirium performance in the context of different dementia severities and subtypes. Eliciting the best methods with low false positives to measure delirium in these contexts should be the other focus of future work. Thirdly, there is a gap in the identification of delirium and catatonia, due to the absence of specific screening programs for catatonia. Given the limited resources available in the healthcare system, there is a need to develop effective screening, identification, and treatment guidelines for the risk of catatonia and delirium. Finally, while some elements of multi-component programs, such as ensuring adequate hydration and nutrition, detecting and treating pain and constipation, avoiding delirium-inducing drugs, and refraining from urinary catheterization, have shown promise in the prevention and management of delirium, few studies have been conducted to demonstrate their efficacy in psychiatric settings. Therefore, intervention studies are needed to assess the effects of multi-component intervention programs on delirium in patients with psychiatric illness.

## Conclusions

Given the high rate of misdiagnosis and the consequences of misdiagnosis of delirium, it is imperative to strengthen the identification of delirium symptoms and modify risk factors in patients with psychiatric illness in psychiatric settings. The close and continuous contact with patients allows clinicians and nurses to identify subtle differences between delirium and psychiatric illnesses with the combination of delirium assessment tools, which will facilitate the management of delirium in psychiatric settings. Additionally, multi-component programs targeting the modifiable risk factors associated with delirium should be introduced into psychiatric clinical practice.

### Electronic supplementary material

Below is the link to the electronic supplementary material.

Supplementary Material 1

## Data Availability

All data generated or analyzed during this study are included in this published article and its supplementary information files.
