# Supplementary material for: Delirium in psychiatric settings: risk factors and assessment tools in patients with psychiatric illness: a scoping review
Source: BMC Nurs. 2024 Jul 8;23:464. doi: 10.1186/s12912-024-02121-6 (PMC11229275; doi:10.1186/s12912-024-02121-6)
Supplement: Supplementary file 1 — Supplementary Material 1 [file 12912_2024_2121_MOESM1_ESM.docx]

**Supplemental Material 1** **Overview of the selected studies**

| **Author(s), year of publication** | **Study location** | **Aim** | **Design** | **Sample** | **Key findings** |
| --- | --- | --- | --- | --- | --- |
| Quispel-Aggenbach, 2021 | Netherlands | To investigate the prevalence and risk factors of delirium. | cross-sectional study | Total, n=444,;  probable delirium, n=85 | Age, prior delirium and infection were independent factors associated with an increased risk of delirium. |
| Patten, 2001 | Canada, | To identify the clinical and pharmacological determinants of delirium. | Case-control study | Delirium prevalent, n=91; developing delirium, n=31, | Medication exposures, lithium, anticholinergic, and antipsychotics were significantly associated with delirium. |
| Ritchie, 1996 | Canada, | To investigated risk factors for delirium among hospitalized psychiatric patients. | Retrospectively study | Total, n=199, delirium, n=29 | Antiparkinsonian and older age were significantly associated with delirium. |
| Bauernfreund, 2023 | UK | Establish factors associated with delirium in a population with severe mental illness. | Retrospective cohort study | Total, n=85,979; delirium, n=1689 | Delirium was associated with older age, bipolar affective disorder, other psychosis, more physical comorbidities, received antipsychotic medication. |
| Ola, 2010 | Nigeria | Examine if any demographic or clinical variables were correlated with delirium. | Prospective survey design | Total, n=264; delirium, n=48 | Physical comorbid, infection, psychotropic medication was associated with the presence of delirium. |
| Patten, 1997 | Canada | Identify risk factors for delirium in psychiatric inpatients. | Prospective cohort study | Total, n=401; delirium, n=9 | Anticholinergic, antipsychotics, electroconvulsive, lithium–anticonvulsant–antipsychotic combination was the potential risk factors for delirium. |
| Friedrich, 2022 | Germany | To estimate whether antidepressants or antipsychotics contribute to the risk of drug-induced delirium (DID). | Cohort study | Total, n=436,565; delirium, n=254 | Tricyclic antidepressants, clozapine, and drugs with antimuscarinic properties were associated with DID. |
| Ma, 2017 | China, | To report duloxetine-and bupropion-related delirium in depressive disorder. | Case report | One case with depressive disorder | Caution should be exercised when using bupropion in combination with other antidepressants. |
| Lertxundi, 2013 | Spain, | To report a case of schizoaffective disorder who developed delirium. | Case report | schizoaffective disorder and multiple sclerosis | The use of levofloxacin in psychiatric patients should be vigilant against the occurrence of delirium. |
| Huang, 1998 | China, | To investigate the reasons, and clinical features of delirium in psychiatric inpatients. | longitudinal study | Total, n=2512, delirium, n=34 | The most common cause of delirium was adverse effects of medication. |
| Figiel, 1991 | U.S | To examine the incidence of ECT-induced delirium in depressed patients. | prospective investigation | Total, n=7 with a diagnosis of Parkinson’s disease. | Structural changes in basal ganglia may predispose individuals to develop delirium during ECT. |
| Patel, 2020 | U.S, | To investigate the correlation between electroconvulsive therapy and lithium ion therapy in the treatment of delirium. | Case-control study | Total, n=64728, ECT + lithium, n=422 | Patients managed with Li + ECT had 11.7-fold higher odds of delirium. |
| Morandi, 2020 | Italy | To investigate the associated factors of delirium subtypes in patients with dementia. | cross-sectional study | Total dementia, n=1057, delirium, n=371 | Hypoactive delirium: venous catheterization, urinary catheterization; Mixed delirium: intravenous catheters, catheters, antipsychotics and antibiotics; Hyperactivity delirium: catheters and antipsychotics. |
| Ali, 2023 | India | To present two cases who developed delirium after receiving a combination of ECT and lithium. | Case report | Two cases with bipolar affective disorder | Combining ECT and lithium to treat acute manic episodes can cause delirium. |
| Barra, 2023 | Spain | To identify the factors predict delirium in a hospitalized psychogeriatric population. | cross-sectional, study | Total, n=1017, delirium, n=445 | Advanced age, physical disability, history of delirium and no use of benzodiazepines were risk factors for developing delirium. |
| Manepalli, 1990 | U.S | To report the prevalence of delirium and urinary tract infection in psychogeriatric unit. | retrospective study | Total, n=407, delirium, n=54 | Urinary tract infection was a potential contributing factor of delirium. |
| Ren, 2023 | China | To investigate the prevalence and risk factors of delirium in psychiatric patients with critical illness. | retrospective study | Total, n=425, delirium, n=143 | Infectious disease, electrolyte disturbance, organic mental disorder and marital status were risk factors of delirium. |
| Suzuki, 1999 | Japan | To examine the causal factors of delirium in elderly patients. | case analysis | Total, n=43, delirium, n=43 | Psychotropic drugs were the predisposing factors of delirium in psychiatric hospital. |
| Yang, 2012 | Korea | To determine the effect of adjuvant light therapy with antipsychotic treatment in delirium. | Randomized group study | Risperidone group, n=16, risperidone with light, n=20 | The risperidone with light therapy group improved the total sleep time and sleep efficiency, which may be a helpful adjuvant treatment for patients with delirium. |
| Youssef, 2023 | U.S | To examine the percentage of delirium in patients treated with ECT plus lithium. | Longitude study | Total, n=210, delirium, n=15 | Advanced age, female, major depressive disorder were the risk factors of drug-induced delirium |
| Arnold, 2005 | U.S | To examine how the delirium, dementia and depression differ. | Review | - | Infection, and urinary tract infection can trigger delirium. Support the patient’s recovery by providing a  stable, structured environment. |
| Dening, 2019 | UK | To enable nurses to differentiate between dementia, delirium and depression. | Review | - | Aged over 65 years was a potential risk factors of delirium. The prevention and management of delirium may overlap and may not be distinct |
| Downing, 2013 | U.S | To recognize the presentations in patient with delirium, dementia and depression. | Review | - | Restrictive furniture, and chemical restraints are associated with delirium. Primary care has a foundation for identification, triage, and treatment in delirium. |
| Edwards, 2003 | U.S | To compare and contrast delirium, dementia, and depression. | Review | - | Common cause of delirium includes infection, antipsychotics, and antidepressants. Awareness of the differences can lead to early identification and treatment. |
| Wilson, 2021 | U.S | To provide an educational review to assist clinicians in identifying and managing delirium. | Case report | - | Detailed evaluation is often required to differentiate such instances of pseudo-delirium from delirium proper. |
| Karmacharya， 2008 | U.S | To examine clinical characteristics of patients with delirium and mania to a psychiatric inpatient unit. | Retrospective study | - | Delirium should be considered in patients with a history of bipolar disorder. |
| Rosen, 1994 | U.S | To distinguish delirious from non-delirious using Delirium Rating Scale (DRS). | Diagnostic study | Total, n=791, delirium，n=70 | DRS score of ≥10 correctly identified delirious patients with a sensitivity of 94% and a specificity of 82%. |
| Trzepacz, 1988 | U.S | To developed a criterion-based symptom rating scale, the DRS. | Diagnostic study | Total, n= 47, delirium, n=20 | The DRS appears to identify delirium and quantitate its severity, and clearly differentiated delirious. |
| Trzepacz, 2001 | U.S | To validate the Delirium Rating Scale-Revised-98 (DRS-R-98) for distinguishing delirious from non-delirious patients. | Diagnostic study | Total, n=68, delirium, n=24 | The DRS-R-98 is a valid and reliable symptom severity scale for delirium against a dementia group and other psychiatric diagnostic groups. |
| Lee, 2011 | Korea | To validate the reliability of the DRS-R-98-K for discriminating delirium from other non-delirious. | Diagnostic study | Total, n=157, delirium, n=69 | DRS-R-98-K is a reliable and valid instrument for diagnosis and severity assessment of delirium. |
| de Negreiros, 2008 | Portugal | To assess the validity and the reliability of the Portuguese version of the DRS-R-98. | Diagnostic study | Total, n=64, delirium, n=27 | The Portuguese version of the DRS-R-98 is an effective tool for evaluating delirium and the severity of delirium. |
| Kim, 2022 | Korea | To explored the inter-rater reliability of the K-DDT-Pro between an expert and a non-expert. | Diagnostic study | Total, n=42, delirium, n=25 | The K-DDT-Pro is a brief and simple tool that usefully screens for delirium in elderly patients. |
| Matsuoka, 2001 | Japan | To assess the reliability and validity of the Japanese Memorial Delirium Assessment Scale (MDAS) in psychogeriatric unit. | Prospective, diagnostic study | Total, n=37, delirium, n=16 | The Japanese version of the MDAS has acceptable reliability for assessing delirium among psycho-geriatric populations. |
| Meagher, 2020 | Ireland | To compare the accuracy of two novel bedside tests with conventional bedside cognitive tests in identifying delirium. | Cross-sectional study | Total, n=180, delirium, n=44 | The Lighthouse test and the Letter and Shape Drawing test (LSD-4) are novel tests with high accuracy for detecting delirium. |
| Hercus and Hudaib, 2020 | Australia | To determine the number of misdiagnosed cases referred to Consultation-Liaison Psychiatry. | Retrospective study | Total, n=584, delirium, n=74 (missing data, n=2) | The predictive algorithm achieved a Area under the curve AUC of 79%, 77% sensitivity and 67% specificity. |
| Baranowski, 2000 | Canada | To evaluates the predictive value of dysgraphia and constructional apraxia for delirium in psychiatric inpatients. | Diagnostic study | Total, n=415, delirium, n=55 | Dysgraphia and constructional apraxia are useful clinical signs of delirium in the psychiatric inpatient population. |
